# Antioxidants for Healthy Skin: The Emerging Role of Aryl Hydrocarbon Receptors and Nuclear Factor-Erythroid 2-Related Factor-2

**DOI:** 10.3390/nu9030223

**Published:** 2017-03-03

**Authors:** Masutaka Furue, Hiroshi Uchi, Chikage Mitoma, Akiko Hashimoto-Hachiya, Takahito Chiba, Takamichi Ito, Takeshi Nakahara, Gaku Tsuji

**Affiliations:** 1 Department of Dermatology, Kyushu University, Maidashi 3-1-1, Higashi-ku, Fukuoka 812-8582, Japan; uchihir@dermatol.med.kyushu-u.ac.jp (H.U.); mchikage@dermatol.med.kyushu-u.ac.jp (C.M.); ahachi@dermatol.med.kyushu-u.ac.jp (A.H.-H.); allheartakita@yahoo.co.jp (T.C.); takamiti@dermatol.med.kyushu-u.ac.jp (T.I.); nakahara@dermatol.med.kyushu-u.ac.jp (T.N.); gakku@dermatol.med.kyushu-u.ac.jp (G.T.); 2 Research and Clinical Center for Yusho and Dioxin, Kyushu University, Fukuoka 812-8582, Japan; 3 Division of Skin Surface Sensing, Department of Dermatology, Kyushu University, Fukuoka 812-8582, Japan

**Keywords:** antioxidants, reactive oxygen species, aryl hydrocarbon receptor, nuclear factor-erythroid 2-related factor-2, phytochemicals

## Abstract

Skin is the outermost part of the body and is, thus, inevitably exposed to UV rays and environmental pollutants. Oxidative stress by these hazardous factors accelerates skin aging and induces skin inflammation and carcinogenesis. Aryl hydrocarbon receptors (AHRs) are chemical sensors that are abundantly expressed in epidermal keratinocytes and mediate the production of reactive oxygen species. To neutralize or minimize oxidative stress, the keratinocytes also express nuclear factor-erythroid 2-related factor-2 (NRF2), which is a master switch for antioxidant signaling. Notably, there is fine-tuned crosstalk between AHR and NRF2, which mutually increase or decrease their activation states. Many NRF2-mediated antioxidant phytochemicals are capable of up- and downmodulating AHR signaling. The precise mechanisms by which these phytochemicals differentially affect the AHR and NRF2 system remain largely unknown and warrant future investigation.

## 1. Introduction

Oxidative stress is defined as an imbalance between the formation of oxidative free radicals and the antioxidant defense capacity of cells of the body [1]. Oxidative stress has been shown in many dermatological diseases, including vitiligo, atopic dermatitis, alopecia areata, photoaging, carcinogenesis, and chemotoxicity [2,3,4,5,6,7,8,9,10,11,12]. Most free radicals in the body exist in the form of reactive oxygen species (ROS). Excessive free radicals impair not only DNA, but also cellular proteins and lipids [9,10]. 

In living cells, ROS are continuously generated as a byproduct of oxidative energy metabolism to make adenosine triphosphate from glucose in mitochondria, by xanthine oxidase for the degradation of purine nucleotides, by nitric oxide synthase to make nitric oxide, and so on [10]. In addition, external stimuli, such as ionizing and ultraviolet (UV) radiation, environmental pollutants, contact allergens, and drugs, are potent inducers of ROS production [9,12,13,14,15]. Inflammatory cytokines are also responsible for ROS generation [9,10,16]. Since the skin is the outermost organ of the body, these oxidative stimulants adversely affect the proper differentiation and barrier function of the skin. One of the major sensors recognizing these stimulants in the skin is aryl hydrocarbon receptor (AHR), which was originally called dioxin receptor [13]. 

The excessive production of ROS should be neutralized or minimized by antioxidants in order to maintain skin homeostasis. The antioxidant molecules, including glutathione, vitamin E, and vitamin C work together with enzymatic antioxidants, such as NAD(P)H:quinone oxidoreductase 1 (NQO1), heme oxygenase-1 (HO-1), and glutathione S-transferase [17]. The induction of these antioxidant enzymes is regulated by nuclear factor-erythroid 2-related factor-2 (NRF2), which is a master switch for antioxidant signaling [13,17]. 

Various phytochemicals and herbal extracts exert their antioxidant properties by activating the NRF2 system in an AHR-dependent or AHR-independent manner in human epidermal keratinocytes [18,19,20,21,22]. Certain antioxidant phytochemicals also upregulate the expression of filaggrin (FLG), which plays a pivotal role in maintaining epidermal barrier function [19,20,21,23].

## 2. Aryl Hydrocarbon Receptor Regulating both Oxidative and Antioxidant Pathways

**Figure 1:**
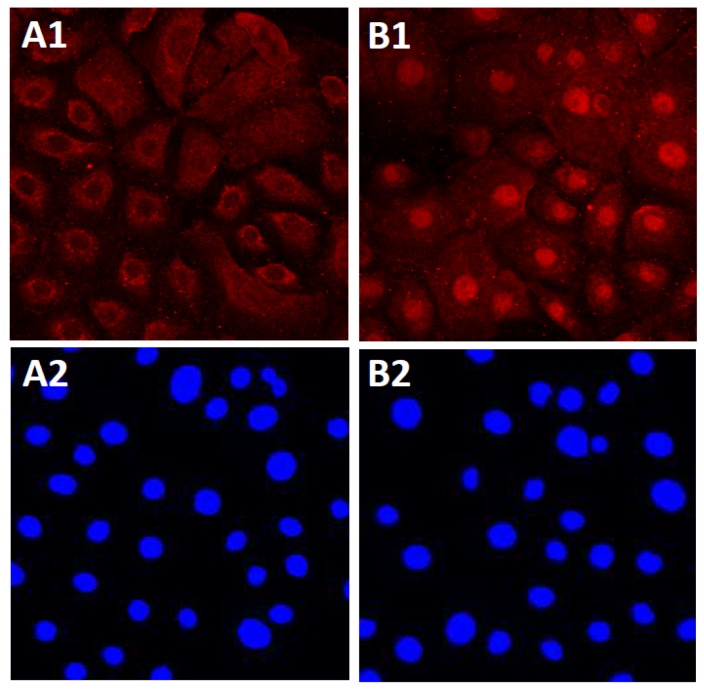
Activation of AHR. In untreated normal human keratinocytes, AHR (red) is mainly located in the cytoplasm (**A1**). Nuclei are stained with 4′,6-diamidino-2-phenylindole (blue, **A2**). In the presence of soybean tar glyteer, AHR is translocated from the cytoplasm to the nucleus (**B1**, red). Nuclei are stained with 4′,6-diamidino-2-phenylindole (blue, **B2**).

AHR is a xenobiotic chemical sensor abundantly expressed in the epidermal keratinocytes [13,24]. Various external and internal ligands, such as dioxins, polycyclic aromatic pollutants, benzo[a]pyrene, phytochemicals, and food metabolites bind to, and activate, AHR [13,24]. Tryptophan photoproduct 6-formylindolo[3,2-b]carbazole (FICZ), generated by UV irradiation, is also known as a high-affinity endogenous ligand for AHR [25,26]. Historically, the signaling pathway of AHR has been elucidated in studies investigating the toxicity of dioxins and polycyclic aromatic pollutants [13,27,28]. Upon ligation by dioxins, the activated AHR translocates from the cytoplasm into the nucleus (Figure 1). This translocated AHR binds to its specific DNA recognition site, namely, xenobiotic-responsive element, and upregulates the transcription of responsive genes, such as cytochrome P450 1A1 (*CYP1A1*) [13,27,28]. CYP1A1 is a member of a multigene family of xenobiotic-metabolizing enzymes [13,27,28]. Besides its physiological role in the detoxification of dioxins, the activity of CYP1A1 can be deleterious because it generates mutagenic metabolites and ROS. FICZ binds to AHR and upregulates the expression of CYP1A1 in an efficient but transient manner; this is because FICZ is rapidly metabolized by CYP1A1 in a feedback mechanism [25,29]. Extensive studies on the function of AHR using AHR-deficient mice have demonstrated that AHR is responsible for most, if not all, of the toxic effects caused by dioxins [30]. 

In addition to oxidative stress, recent studies have demonstrated that the AHR system mediates antioxidative and protective signaling in response to certain ligands, such as flavonoids, herbal medicines, and azoles [16,18,19,21,31,32,33]. For example, ligation of AHR by ketoconazole induces the nuclear translocation of AHR without producing ROS. Instead, it activates the NRF2-NQO1 pathway, which protects cells from ROS-induced oxidative damage [16]. There are several types of AHR ligands. Dioxins, benzo[a]pyrene, and other polycyclic aromatic pollutants bind to AHR with high affinity and induce tremendously high *CYP1A1* expression with damaging ROS production [14,30]. Although subsequent NRF2 activation does occur after AHR ligation by dioxins [34], the oxidative stress overwhelms the antioxidant protection in response to these hazardous compounds [14,31]. A plethora of beneficial and antioxidant phytochemicals, such as cynaropicrin, activate the AHR-NRF2 signaling pathway without any appreciable production of ROS [18]. On the other hand, the antioxidant cinnamaldehyde instead inhibits AHR activation. However, it potently activates the NRF2 pathway and exerts antioxidant activity in an AHR-independent manner [22]. The precise mechanisms by which these chemicals differentially affect the AHR-NRF2 system remain largely unknown. Since AHR forms a molecular complex with Hsp90, XAP2, and p23 in the cytoplasm and with AHR nuclear translocator (ARNT) in the nucleus, these partner molecules may potentially define the oxidative and antioxidant outcome [30]. 

Another intriguing paradigm for AHR signaling is its enhancing effects on epidermal barrier function. The barrier function is significantly disrupted in *Ahr*-null mice, indicating that AHR plays a pivotal role in skin barrier integrity [35]. FLG is one of the major components of barrier proteins [23,36]. Loss-of-function mutation of *FLG* causes dry skin and is critically involved in the pathogenesis of atopic dermatitis [37,38,39]. Antioxidant folk medicines such as *Houttuynia cordata* extract and *Opuntia ficus-indica* extract potently activate the AHR-NRF2 pathway and upregulate *FLG* expression [20,21]. The bifunctional (antioxidant and barrier-protection) properties of these folk remedies are particularly promising for maintaining the health of the skin.

## 3. Nuclear Factor-Erythroid 2-Related Factor-2, a Master Transcription Factor for Inducing Antioxidant Enzymes

**Figure 2:**
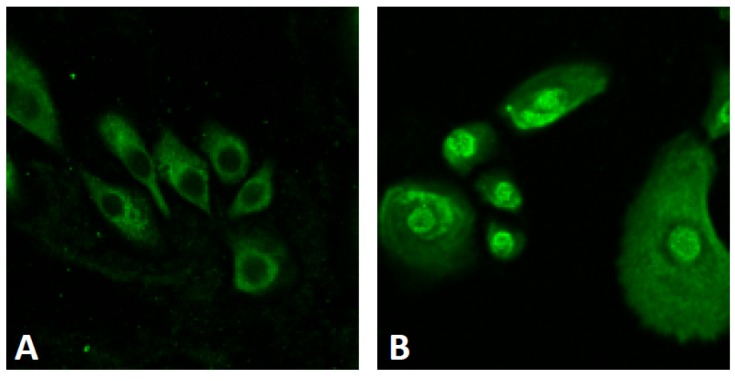
Activation of NRF2. In untreated normal human keratinocytes, NRF2 (green) is mainly located in the cytoplasm (**A**); *Opuntia ficus-indica* extract activates NRF2 and induces its cytoplasmic to nuclear translocation (**B**).

The transcription factor NRF2 is a master switch for inducing antioxidant enzymes and is expressed in epidermal keratinocytes at high levels [16,17,31,32]. The antioxidant enzymes downstream of NRF2 include NQO1, HO-1, glutathione S-transferase, UDP-glucuronosyltransferases, epoxide hydrolase, glutathione reductase, thioredoxin reductase, catalase, and superoxide dismutase. NRF2 also activates the transcription of non-enzymatic antioxidant protein genes, such as thioredoxin and ferritin [17]. Under physiological conditions, the level of NRF2 in the cytoplasm is regulated by the formation of the NRF2-KEAP1-CUL3 complex [17]. KEAP1 binds to NRF2 and, therefore, directly inhibits its activity, resulting in simultaneous NRF2 ubiquitination catalyzed by CUL3. However, the oxidative condition in the cell leads to the oxidation of cysteine residues in the KEAP1 molecule, changing its conformation and causing dissociation of NRF2 from the complex. This free NRF2 is translocated to the nucleus and initiates the transcription of antioxidant genes (Figure 2) [17].

In *Nrf2*-null mice, UVB-induced sunburn reaction became significantly stronger and longer-lasting with a reduction of inducible HO-1 expression compared with that in wild-type mice [40,41]. In addition, mutation of the NRF2 gene has been suggested to be oncogenic in some squamous cell carcinoma cases [42]. The expression of NRF2 protein is downregulated in human malignant skin tumors [43]. On the other hand, excessive antioxidant activity does hamper the epidermal barrier function. K5-Cre-Nrf2 transgenic mice generated by Schäfer et al. were found to express high levels of constitutively-active Nrf2 in the epidermis together with the overexpression of Nqo1 and other antioxidative enzymes. Unexpectedly, their skin is dry with hair loss, scaling, epidermal acanthosis, and hyperkeratosis [44,45]. The Nrf2 transgenic mice gradually developed severe chloracne-like lesions, which are highly reminiscent to the patients with chloracene/metabolizing acquired dioxin-induced skin hamartomas [45]. Furthermore, the Nrf2 activation promotes the human papilloma virus-induced or arsenite-induced carcinogenesis by upregulating the survival and proliferation of transformed keratinocytes [46,47]. These studies stress the importance of an appropriate balance between oxidative and antioxidative processes in maintaining epidermal homeostasis.

## 4. Phytogenic Antioxidants

**Figure 3:**
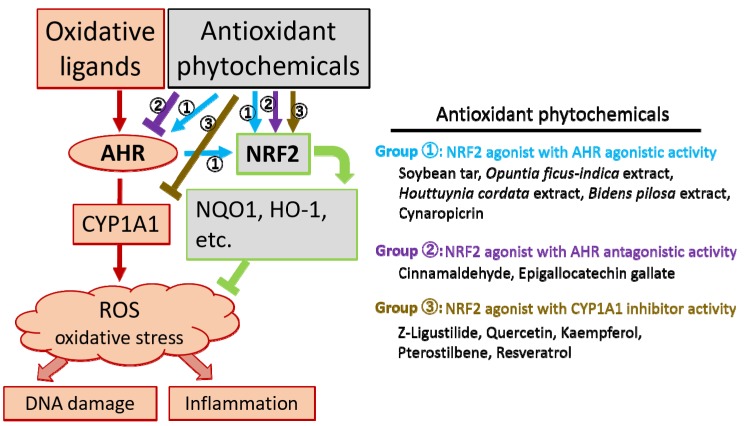
Antioxidant phytochemicals differentially modulate aryl hydrocarbon receptor (AHR), cytochrome P450 1A1 (CYP1A1) and nuclear factor-erythroid 2-related factor-2 (NRF2). Oxidative ligands, such as ultraviolet radiation, dioxins, and environmental polycyclic pollutants, activate the AHR and CYP1A1 system, which generates reactive oxygen species (ROS) and causes DNA damage and inflammation. Antioxidant phytochemicals exert their antioxidant capacity by activating NRF2, which is a master transcription factor for the induction of antioxidant enzymes such as NAD(P)H: quinone oxidoreductase 1 (NQO1) and heme oxygenase-1 (HO-1). These antioxidant enzymes neutralize or minimize ROS production. Antioxidant phytochemicals are categorized into at least three groups based on their capacity for up- and downmodulating AHR and CYP1A1. Group 1 contains NRF2 agonists with AHR agonistic activity (①). Group 2 contains NRF2 agonists with AHR antagonistic activity (②). Group 3 contains NRF2 agonists with CYP1A1 inhibitor activity (③).

Most phytogenic antioxidants are plant phenolic compounds. Approximately 8000 different structures of plant phenolics are known [48]. These phenolic compounds are classified into flavonols, flavones, flavonones, flavanols, isoflavones, anthocyanidins, hydroxycinnamic acids, hydroxybenzoic acids, tannins, stilbens, and lignans [48]. Some phytochemicals have been shown to bind to AHR with different affinities [49,50,51]. Below, we discuss in detail several phytogenic antioxidants, with special reference to AHR and NRF2 signaling in epidermal keratinocytes (Figure 3).

### 4.1. NRF2 Agonist with AHR Agonistic Activity

#### 4.1.1. Soybean Tar

Soybean tar glyteer has been widely used for the treatment of various inflammatory skin diseases in Japan since 1924, as an alternative to coal tar remedy [19]. As has been demonstrated for coal tar [52], glyteer inhibits the ROS production by benzo[a]pyrene and TNFα via NQO1 upregulation [19]. The antioxidant activity of soybean tar is mediated by the AHR-NRF2 pathway because it was shown to be attenuated by transfection with small interfering RNA against either AHR or NRF2 [19]. In addition, glyteer upregulates FLG expression in an AHR-dependent manner [19].

#### 4.1.2. Opuntia Ficus-Indica Extract

**Figure 4:**
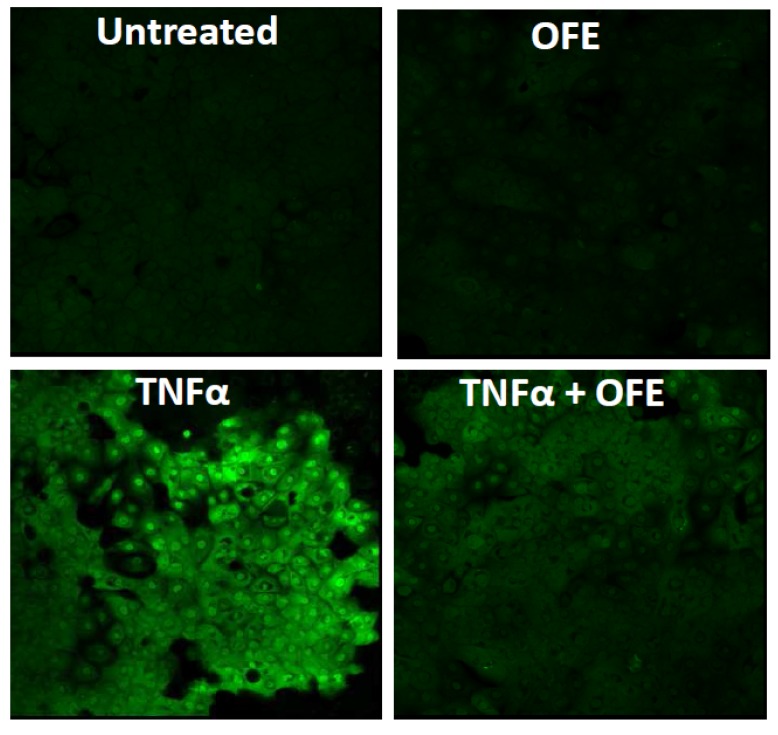
Antioxidant activity of *Opuntia ficus-indica* extract (OFE). Reactive oxygen species (ROS) are visualized with dichloro-dihydro-fluorescein diacetate staining (green). The production of ROS is not active in the untreated or OFE-treated human keratinocytes. Tumor necrosis factor α (TNFα) induces ROS production, which is significantly inhibited by OFE (TNFα + OFE).

*Opuntia ficus-indica* is a cactus species widely used as an anti-inflammatory, antilipidemic, and hypoglycemic agent [21]. Studies have suggested that its extract can downregulate oxidative stress via benzo[a]pyrene and TNFα (Figure 4). Its potent antioxidant activity is also mediated by the AHR-NRF2-NQO1 pathway [21]. *Opuntia ficus-indica* extract also stimulates AHR and upregulates FLG expression [21].

#### 4.1.3. *Houttuynia cordata* Extract

*Houttuynia cordata*, which is called “dokudami” in Japanese, is an aromatic medicinal herb that has been traditionally eaten as a folk medicine for various ailments, such as diabetes, obesity, cough, fever and skin diseases, in Asia [20]. Similar to glyteer and *Opuntia ficus-indica* extract, *Houttuynia cordata* extract inhibits the ROS production by benzo[a]pyrene and TNFα via the AHR-NRF2-NQO1 pathway [20]. 

#### 4.1.4. *Bidens pilosa* Extract

*Bidens pilosa* is a tropical weed that grows widely in tropical and subtropical regions. This plant is used in various folk medicines and as a popular ingredient in herbal tea for its blood-pressure-lowering, liver-protective and hypoglycemic effects [53]. In the therapeutic guidelines for vasculitis and vascular disorders of the Japanese Dermatological Association, *B. pilosa* extract is recognized as an effective remedy for the treatment of livedo vasculopathy [53]. This extract potently inhibits the ROS production of endothelial cells by upregulating NRF2 and NQO1, which are abrogated by the knockdown of AhR or Nrf2 [53].

#### 4.1.5. Cynaropicrin

Artichoke (*Cynara scolymus*) is one of the most ancient plants grown in the world and has been used as a folk medicine in the treatment of hepatitis, hyperlipidemia, obesity and dyspeptic disorders [18]. Cynaropicrin, a sesquiterpene lactone, is one of the major bioactive phytochemicals in artichoke extract [18]. The ROS production in UVB-irradiated keratinocytes is significantly downregulated by cynaropicrin [18]. The antioxidant activity of cynaropicrin is AHR-NRF2-dependent and inhibits the ROS production by benzo[a]pyrene and tumor necrosis factor α (TNFα) [18]. However, the AHR agonistic potency of cynaropicrin is very weak compared with those of soybean tar, *Opuntia ficus-indica* extract, *Houttuynia cordata* extract, and *Bidens pilosa* extract [19,20,21,53].

### 4.2. NRF2 Agonist with AHR Antagonistic Activity

#### 4.2.1. Cinnamaldehyde

Cinnamaldehyde (3-phenyl-2-propenal) is the major constituent of the bark of *Cinnamomum cassia*, and it is known to have various biological activities including anti-inflammatory and anti-bacterial properties [22]. Cinnamaldehyde exerts its antioxidant activity via NRF2 and HO-1 expression and downregulates benzo[a]pyrene-induced oxidative stress [22]. However, cinnamaldehyde is unable to activate AHR, but instead significantly inhibits its action [22], which is in sharp contrast to cynaropicrin, soybean tar, *Opuntia ficus-indica* extract, *Houttuynia cordata* extract, and *Bidens pilosa* extract [19,20,21,53]. The dual functions of cinnamaldehyde, namely, inhibition of AHR and activation of NRF2, may be particularly beneficial for the treatment of intoxication of environmental pollutants such as dioxin [22].

#### 4.2.2. Epigallocatechin Gallate

**Figure 5:**
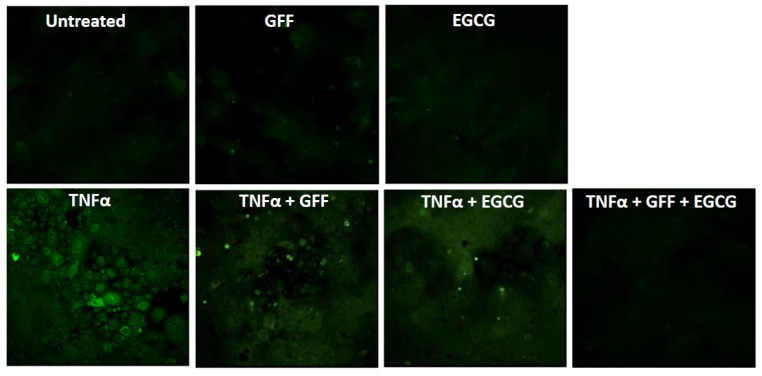
Synergistic antioxidant activity of *Galactomyces* ferment filtrate (GFF; 0.1%) and epigallocatechin gallate (EGCG; 10 μM). Reactive oxygen species (ROS) are visualized with dichloro-dihydro-fluorescein diacetate staining (green). The production of ROS is very slight in the human keratinocytes treated with medium control (untreated), a low concentration of GFF, or a low concentration of EGCG. Tumor necrosis factor α (TNFα) induces ROS production, which is weakly inhibited by a low concentration of either GFF (TNFα + GFF) or EGCG (TNFα + EGCG). The ROS production by TNFα is markedly downregulated by the simultaneous addition of GFF and EGCG (TNFα + GFF + EGCG).

The green tea flavonoid epigallocatechin gallate upregulates Nrf2 and HO-1 expression [54] and inhibits AHR action [55,56]. It has been increasingly used for cosmetic purposes [57]. *Galactomyces* fermentation filtrate is also an AHR-stimulant generally used in cosmetics [58]. Interestingly, epigallocatechin gallate and *Galactomyces* fermentation filtrate exhibit synergistic antioxidant activity against TNFα-induced ROS production (Figure 5). The antagonistic or agonistic potency of epigallocatechin gallate for AHR may vary depending on the cell type [59].

### 4.3. NRF2 Agonist with CYP1A1 Inhibitor Activity

#### 4.3.1. Z-Ligustilide

Z-Ligustilide is the major bioactive phthalide of *Cnidium officinale* and *Angelica acutiloba*, which are widely used in folk medicine in East Asia [60,61]. Z-Ligustilide ameliorates UVB-induced oxidative stress and inflammatory cytokine production through upregulation of the NRF2/HO-1 pathway and the suppression of NF-κB signaling [61]. Z-Ligustilide is not an AHR ligand, but it significantly inhibits benzo[a]pyrene-induced CYP1A1 expression via NRF2 activation [60].

#### 4.3.2. Quercetin, Kaempferol, and Pterostilbene

Quercetin, kaempferol, and pterostilbene are abundant in berries and have potent antioxidant capacity, which is also mediated by the activation of NRF2 [62]. They also exhibit synergistic antioxidant activity when added in combination at appropriate concentrations [62]. Quercetin is not a direct ligand for AHR [26]. It sustains and enhances the ligand activity of AHR, such as FICZ, by inhibiting the CYP1A1-mediated degradation of ligand [26].

#### 4.3.3. Resveratrol

Resveratrol is a widely-available polyphenol found in red wine and other sources that are thought to have health benefits [63]. It is also a potent NRF2 activator and shows antioxidant activity [63]. In addition, it activates sirtuin 1 and AMP-activated protein kinase, an enzyme that initiates autophagy [63]. Resveratrol inhibits the proliferation of human keratinocytes via sirtuin 1 activation [64]. Like quercetin, resveratrol enhances the activity of endogenous FICZ by inhibiting the CYP1A1-mediated degradation of AHR ligands [26]. 

## 5. Conclusions

Skin is inevitably exposed to UV rays and environmental pollutants. To a greater or lesser extent, these external stimulants induce oxidative stress and accelerate skin aging, leading to skin inflammation and carcinogenesis [13,24]. AHR is a capricious receptor that senses various chemical compounds and plays an essential role in photo-induced and chemically-induced oxidative stress [13,19,24,25]. Various antioxidant phytochemicals activate the NRF2 transcription factor and upregulate a series of antioxidant enzymes that neutralize the oxidative stress and protect the keratinocytes from oxidative damage [13,17]. Notably, there is finely-tuned crosstalk between AHR and NRF2, which mutually increase or decrease their activation states. Many NRF2-mediated antioxidant phytochemicals are capable of modulating AHR signaling. There are at least three groups of antioxidant phytochemicals when we categorize their capacity to modulate NRF2, AHR, and CYP1A1 (Figure 3). However, the modulatory capacity of phytochemicals on these three mutually related molecules may be cell-type-specific and concentration-dependent; therefore, the categorization may vary depending on the experimental protocol. The precise mechanisms by which these phytochemicals differentially affect the AHR and NRF2 system remain largely unknown and warrant future investigation.
